# Detection of skin wrinkles and quantification of roughness using a novel image processing technique from a dermatoscope device

**DOI:** 10.1111/srt.13335

**Published:** 2023-05-25

**Authors:** Gustavo Carlos da Silva, Miguel B. Barbosa, Flávio B. Camargo Júnior, Patrícia L. Moreira, Rejane Werka, Airton A. Martin

**Affiliations:** ^1^ Science and Technology Institute University Brazil São Paulo São Paulo Brazil; ^2^ DermoProbes Research and Development LTDA São José dos Campos São Paulo Brazil; ^3^ CHEMYUNION INC Sorocaba São Paulo Brazil; ^4^ i9Magistral São Paulo São Paulo Brazil

**Keywords:** efficacy evaluation, image processing, skin, wrinkles

## Abstract

**Objective:**

Cutaneous relief analysis is crucial in the development of new skincare products, as well as in the evaluation of dermatological treatments. The analysis can be performed by qualitative or quantitative methods. We propose a new algorithm to detect wrinkles and quantify skin roughness by image processing from a dermatoscope.

**Methods:**

A clinical study was carried out with 33 research participants, and images were collected with the dermatoscope and PRIMOS equipment for wrinkle evaluation at two different times: Day 0 (D0) and 45 days (D45) after the use of a dermocosmetic product. Later, a new algorithm was developed to detect wrinkles in the acquired images by applying filters and image transformations that generate a segmented image highlighting the wrinkles. A roughness calculation method is proposed from the pixels belonging to wrinkles.

**Results:**

Correlation between the values obtained by the PRIMOS equipment and the proposed system was verified. No correlation was found for data obtained at D0; however, there was correlation at time D45 by Spearman's similarity coefficient. By comparing roughness between times D0 and D45, the treatment was statistically significant for both PRIMOS and the proposed methodology data.

**Conclusion:**

The wrinkle detection algorithm, in addition to the roughness calculation, demonstrated a sensitivity comparable to the PRIMOS system in evaluating the effectiveness of the dermocosmetic treatment.

**Significance:**

Considering the simplicity of the dermatoscope design compared to other established devices such as PRIMOS, the proposed system is promising as an alternative for dermatological evaluations.

## 1 INTRODUCTION

Wrinkles are important markers of the time effects on human skin. They are objects of study in clinical research aimed at developing cosmetic products capable of minimizing their effects. Moreover, they are studied in dermatological clinics where medical assistance is desirable to propose treatments aimed at improving the appearance of the skin.

The task of comparing wrinkles can be performed subjectively, for which an experienced professional trained according to consolidated classifications, such as Fritzpatrick or Glogau, is required.^1^, ^2^ Another way of assessing wrinkles is by using devices that can quantify them with values that may indicate improvement or worsening of the analyzed area. These techniques are mostly based on image analysis.^3^

The Glogau scale is a wrinkle classification scale proposed in 1997, in which wrinkle levels are divided into four groups according to depth and size. Group I has minimal wrinkles, usually presented in people aged from 20 to 30 years. Group II has smooth skin, but with the appearance of lines when moving, usually presented in people aged from 30 to 40 years. Group III represents visible wrinkles, even without movement or expressions. Finally, Group IV consists of static wrinkles with great depth, presented mainly in individuals over 60 years of age.^4^ Despite the possibility of age classification on the Glogau scale, it is not a rule, as other factors may interfere with the skin's appearance, such as diet or exposure to sunlight.^5^ The Glogau scale may not be efficient in analyzing the improvement or worsening of an aesthetic procedure or the effectiveness of a product, as the treatment's efficiency may not be enough to cause a change among Glogau's groups. Hence, it is crucial to use objective evaluation techniques that can provide quantitative values.

Analysis techniques for image feature extraction have made great progress in skin‐related studies. One of the most established systems is PRIMOS (Canfield, Inc.), which was developed to provide quantitative values for studies and understand the micro‐relief of human skin. This device can measure the depth of furrows, pores, and wrinkles on the skin. The name PRIMOS is an abbreviation for its operating principle, **P**haseshift **R**apid **I**n vivo **M**easurement **O**f the **S**kin, which basically consists of the projection of parallel light strips with minimum differences in angulation, all carried out by micromirrors as described by Jaspers et al.^6^ Its operation is based on the use of a light incidence technique on the skin surface at different angles by micromirrors. This technique is known as fringe projection, which highlights the issues of the analyzed relief. The PRIMOS system is considered the gold standard of skin relief analysis techniques.^7^ Other devices can be cited for skin relief analysis, such as VisioScan VC98 (Courage Kasaka) and the VISIA system (Canfield, Inc.). VisioScan VC98 is a video dermatoscope that has a UVA‐light camera with high resolution based on SELS (Surface Evaluation of the Living Skin) developed by Tronier et al.^8^ While VisioScan VC98 can provide different parameters from the grayscale image, the VISIA system performs skin analysis using multispectral images and presents detailed results of different skin characteristics, in some specific models in combination with PRIMOS technology.^9^

Several digital microscopes are available for use as low‐cost dermatoscopes, which can capture high‐resolution images with magnification levels that reach up to 100 times. However, most of the available software with these devices does not demonstrate reproducibility or assertiveness, and there is no scientific evidence of the provided quantitative values. Suprijanto et al. proposed a method for evaluating skin roughness from digital dermatoscope images using Fourier transform filters.^10^ Image analysis from digital cameras is also used to verify the quality of the skin's relief. Several articles demonstrate methods for wrinkle detection and counting, as well as age determination of individuals based on wrinkle types.

Computer vision comprises the application of mathematical techniques to image processing to obtain specific features according to the application's interest. There are several ways to detect objects or features in an image. Considering the context of this work, focused on wrinkle identification, segmentation becomes crucial because it is possible to identify only the pixels that correspond to the desired region.^11^ Among the most applied methods for segmentation using classical computing techniques, there is the use of histogram processing, edge detection, and pixel clustering. For all these techniques, the focus is on verifying the similarity of neighboring pixels and the consequent decision to separate the different groups according to a threshold.^12^

Several authors have proposed algorithms that show skin features, especially wrinkles. Yap et al. summarized the main algorithms for detecting and reconstructing facial skin wrinkles, among which Cula et al.’s method can be highlighted.^13^, ^14^ At Cula's, the wrinkle index (WI) was defined, which is the product of the wrinkle depth and width, where width is defined by the image's first‐order derivative, and depth is the response of the image's Gabor filter. Another two methods that use the Hessian matrix to determine wrinkles are Hybrid Hessian Filter and Hessian Line Tracking.^15^, ^16^

Recently, with advances in machine learning, new techniques for applying neural networks to image segmentation have been developed, including for skin image analysis, as demonstrated by Rew et al.,^17^ who developed a feature extraction method for images from dermatoscopes through the application of convolutional neural networks (CNNs). The effectiveness of neural networks depends on the number of samples available for network training. The greater the number of examples, the better the performance of a network in solving the proposed problem.

Roughness measurements are important in the analysis of surfaces to verify differences related to relief. The greater the value of the differences between valleys and peaks in a given area, the greater its roughness.^18^ ISO 4287 defines roughness parameters such as arithmetic mean roughness (*R*_a_), root mean squared roughness (*R*_q_), and maximum surface roughness (*R*_max_).^19^ In industry, these parameters are widely used to evaluate material surfaces' quality. The primary parameter for calculating these quantities is the depth measurement of the surface under analysis, typically measured in micrometer.^20^

As human skin also presents irregularities, several authors adapted these parameters to assess skin roughness.^21^ This adaptation includes the main parameter provided by the PRIMOS system, which calculates the average roughness of a defined region. PRIMOS can calculate the parameter *R*_a_.^22^ Askaruly et al. proposed the development of a device that uses optical coherence tomography (OCT) and compared the results to the data obtained using the PRIMOS system in silica and in a clinical study, with OCT showing a lower standard deviation and more reliability than PRIMOS.^23^

Even though PRIMOS is considered the gold standard, it still has a high cost, which limits access to the technology at large clinics, some universities, and research institutes. The low cost of a dermatoscope and the possibility of bringing skin analysis to more clinics and researchers with scientific validation motivate this work. Therefore, we proposed the segmentation of wrinkle areas through the application of digital filters and the development of a mathematical calculation that maximizes the areas of wrinkles, along with a new approach to quantify skin roughness, as well as a comparison with PRIMOS results.

## 2 MATERIALS AND METHODS

### 2.1 Dermatoscope

**FIGURE 1:**
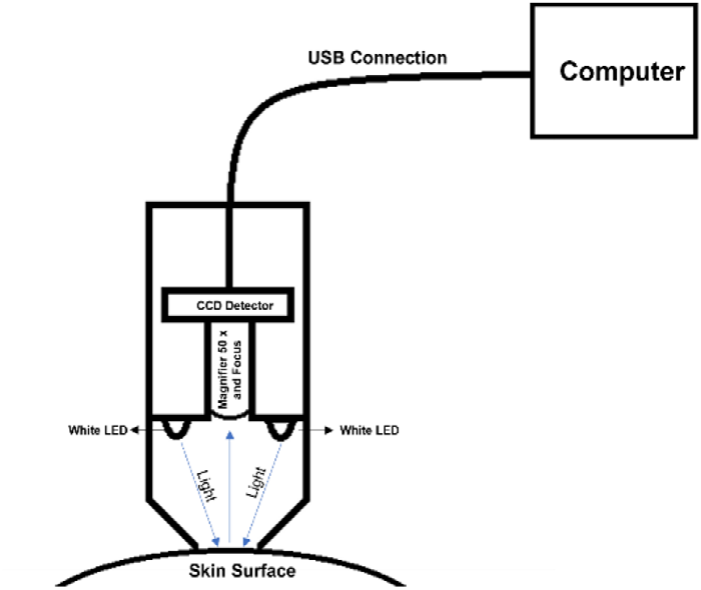
Dermatoscopy representation. The main parts are indicated.

In the system development, a dermatoscope consisting of a USB camera microscope device was used, which can provide images with an optical magnification of 50 times and a resolution of 1600 × 1200 pixels, covering an area of 7 × 6.5 mm. The device is equipped with high‐brightness white LEDs that illuminate the focal point where the image is obtained. Figure 1 shows a representation of the device used. The device has a dark chamber with an opening at the point where the skin must be positioned. Upon activation, the LEDs emit white light toward the skin, illuminating it. The light is captured by the magnifying lens, which is adjusted to focus on the point where the skin surface is positioned. This light is then directed to the CCD detector, which captures the image that is directed through a specific electronic circuit. Finally, the images are available on a computer through the USB connection.

### 2.2 PRIMOS

The comparative study was carried out using the PRIMOS Lite device. The PRIMOS device can provide different quantitative values according to Table 1,^24^ with the *R*_a_ parameter being the most commonly used to evaluate wrinkles. It can be calculated by considering the entire measured area or by delimiting lines at interest points in the processing software available, in conjunction with the PRIMOS system.

**TABLE 1:** PRIMOS parameters.

**parameter**	**Unit**
Wrinkle count	nd
Average depth of wrinkles	µm
Deepest wrinkle	µm
Total wrinkle area	mm^2^
Total wrinkle volume	mm^2^
Total form factor wrinkles	nd
Total length of wrinkles	mm
*R*_a_	µm
*R*_y_	µm
*R*_z_	µm

*Note*: Units denoted by “nd” are non‐dimensional.

### 2.3 Image processing

Digital images are essentially three‐dimensional matrices that contain information about the colors of pixels obtained from a scene. The most common and widely used standard is RGB (red, green, and blue), which was used in this work, and can represent colors by combining the intensity of the three colors. The values for each component are 16‐bit values that range from 0 to 255, where 0 represents the absence of that component and 255 represents the maximum intensity of that color. By processing this matrix, mainly by examining the variation of these values for each pixel and its neighbors, it is possible to extract various types of information from images.^25^

From the images obtained by the dermatoscope, it is possible to observe that the regions representing wrinkles are darker, as they are further from the light source, causing a smaller amount of light to return to the camera as pixels, in comparison to pixels from regions that do not possess wrinkles and are closer to the lens.

**FIGURE 2:**
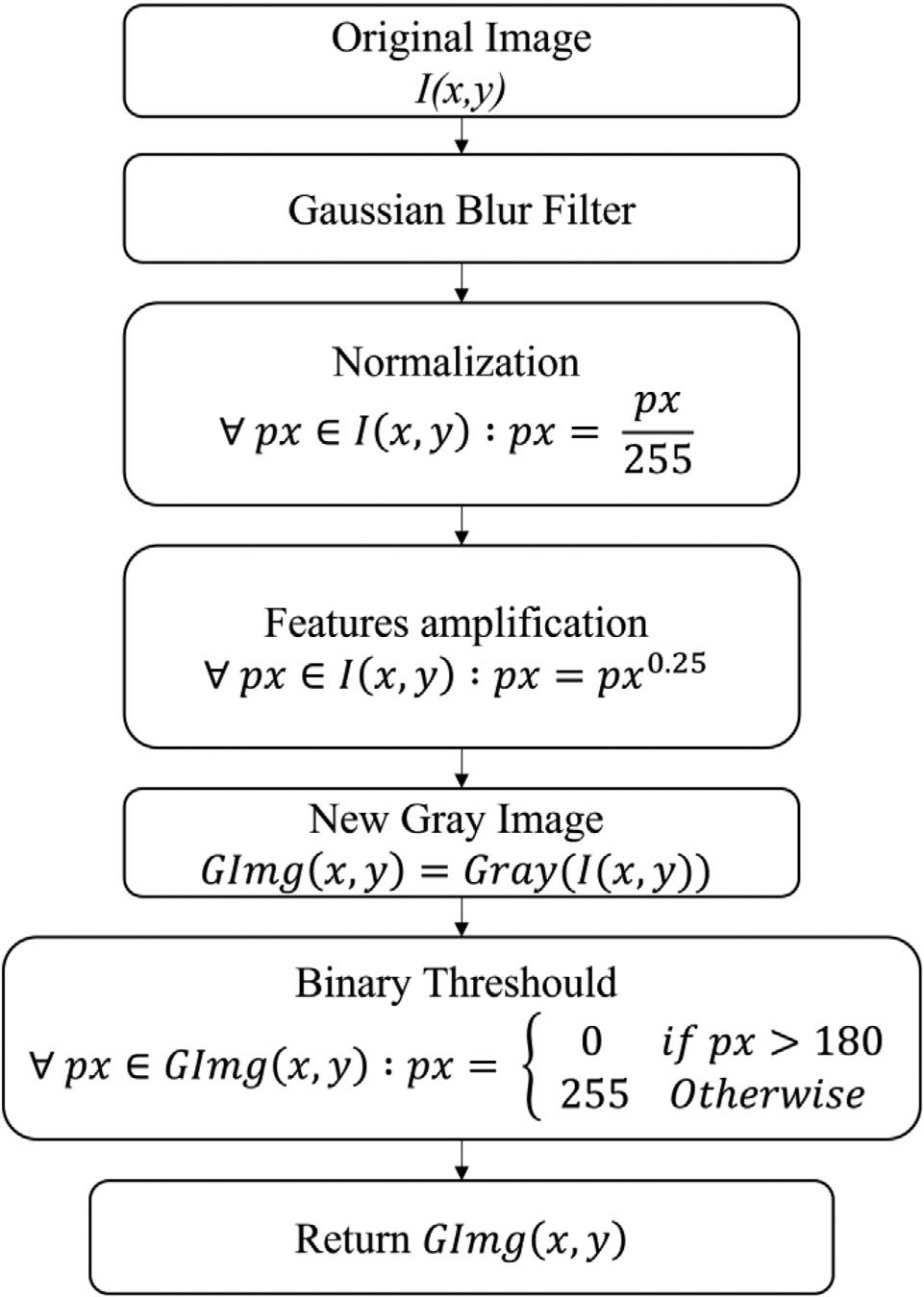
Flowchart of the proposed algorithm with some applied equations.

The first attempt to determine wrinkle areas was performed by converting the original image to a grayscale image with only a 16‐bit color channel, where 0 represents black and 255 represents white, with shades of gray in between these limits.^26^ From the grayscale image, a threshold value was established, and values below it were considered valleys, while values above it were considered peaks, which depend on the analyzed skin tone and the amount of light captured by the camera. Due to the difficulty of directly detecting wrinkles from the grayscale image, the algorithm shown in Figure 2 was developed.

The proposed algorithm's first step consists of applying a Gaussian blur filter to the original image using a 37 × 37 kernel.^27^ The purpose of this filter is to homogenize the light effects in the image and remove small artifacts. The second step involves normalizing the image on a scale from 0 to 1 for all channels by dividing each pixel by the maximum value of 255.^28^ Feature amplification is then performed on the normalized image, which involves raising each pixel value to an exponent. The optimal exponent value was determined to be 0.25 through experimentation. This results in an image where dark tones become darker, approaching 0, and lighter tones become lighter, approaching 1. This characteristic is desirable as wrinkles are determined by the dark areas, which are now even more evident. Hair detection, which generates similar tones to wrinkles, was a point of attention during the first tests of the algorithm. With feature amplification, the hair are no longer segmented in the result. After feature amplification, a new grayscale image is generated, which then passes through a binary threshold filter where pixel values above 180 are assigned the value 0, and below that, the value 255. This generates a black and white image where white pixels represent wrinkle corresponding regions.

It is important to note that the proposed segmentation not only identifies the deeper regions of wrinkles but also covers the adjacent areas at the wrinkle's edges, corresponding to shallower areas of the valley until the skin plateau is reached. The development of all the image processing algorithms was carried out using the Python programming language in conjunction with the OpenCV computer vision library.^29^, ^30^

### 2.4 Roughness quantification

In 2D images, such as those obtained by the dermatoscope device used in this study, it is not possible to measure depth in microns. However, different shades of gray can be observed, with deep parts appearing darker and shallow areas lighter, as mentioned before. With this in mind, a new approach is proposed for calculating the average roughness of a 2D image, focusing especially on the regions where wrinkles are detected. The method consists of calculating the average roughness (*R*_a_) from the original image converted into a grayscale image, considering only pixels belonging to the wrinkle regions detected by the algorithm presented here, and then dividing by the total image area. The value of *R*_a_ is shown in Equation (1).

(1) $$R_{a}=\frac{1}{wh}\;\sum_{i\;=\;0}^{w}\sum_{j\;=\;0}^{h}W_{p}\left(G\left(i,j\right)\right)$$

(2) $$W_{p}\;\left(G\left(i,j\right)\right)=\left\{\begin{array}{c} G\left(i,j\right)\;if\;G\left(i,j\right)\in Wrinkle\\ 0\;\;Otherwise \end{array}\right.\;$$

The *w* value represents the image's width in number of pixels, *h* represents the height, and *G* represents the original image converted into grayscale. For each pixel, its belonging to the group of pixels that make up the detected wrinkle's area is analyzed. This evaluation is performed by the *W*_p_ (wrinkle pixels) function defined in Equation (2), which returns the pixel's value under analysis in case it belongs to the wrinkle area or 0 otherwise. During the development, a modified way of implementing the *R*_q_ calculation was emphasized in the wrinkle area and is described in Equation (3). The parameters considered are the same as those used in the *R*_a_ calculation.

(3) $$R_{q}=\sqrt{\frac{\sum_{i=0}^{w}\sum_{j=0}^{h}{\left(W_{p}\left(G\left(i,j\right)\right)\right)}^{2}}{wh}}\;$$

As the total image size obtained by the dermatoscope is displayed in millimeters and considering the maximum resolution of the image being 1600 × 1200 pixels, it is possible to calculate the area corresponding to each pixel, which is 0.203 × 10^−4^ mm^2^. This makes it possible to calculate the wrinkle area parameter (*W*_a_), consisting of the multiplication of the pixel area by the number of pixels present in the wrinkle area, as described in Equation (4).

(4) $$W_{a}=\;\mathrm{count}\left(px\in \mathrm{Wrinkles}\right)*0.203\times 10^{-4}$$

### 2.5 Research subjects

Total 33 research participants, of all genders and with varying degrees of wrinkles, aged between 19 and 75 years, were selected for this clinical study. The objective of the study was to evaluate the conditions of the wrinkles before and after the use of a dermocosmetic, using both PRIMOS and dermatoscope devices. All participants were recruited and instructed on the procedures, and they signed an informed consent form that was submitted and approved by the research ethics committee (52218121.7.0000.5514).

**FIGURE 3:**
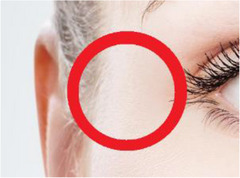
Representation of the periorbital region, in this case highlighted in red next to the right eye.

During the first visit to the study center, images were collected using both PRIMOS and dermatoscope devices at the periorbital region, as shown in Figure 3. After the data collection, participants were given a dermocosmetic and were properly instructed on how to use it by a trained technician from the study center. The dermocosmetic was applied to the periorbital region once a day for 45 consecutive days. On the 46th day, participants returned to the study center for new image collection using both PRIMOS and dermatoscope devices.

**FIGURE 4:**
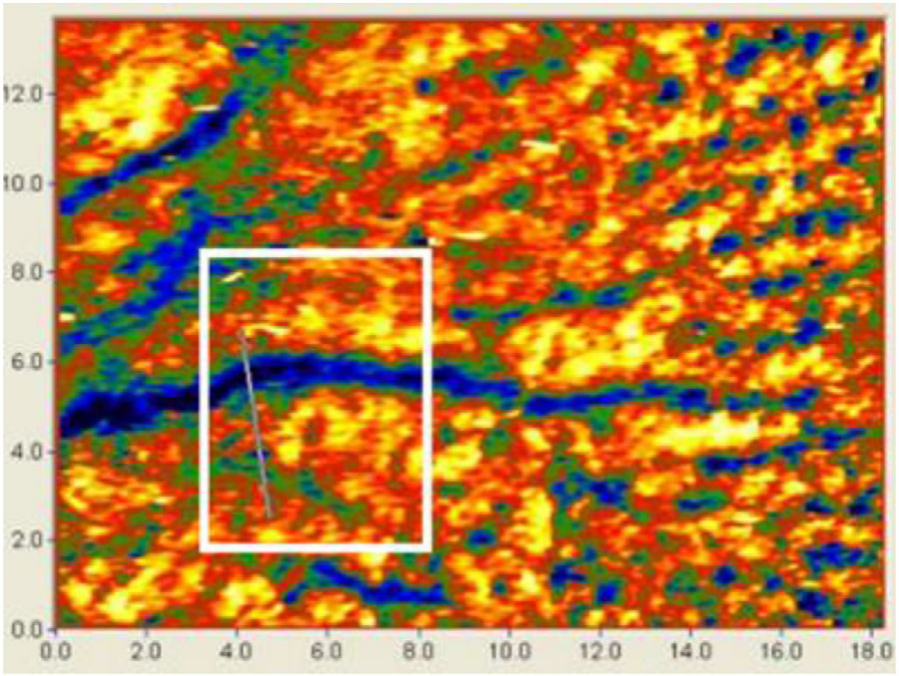
Selection of the area to be analyzed in the PRIMOS software.

Images obtained using the PRIMOS system were analyzed using its specific software. Wrinkle demarcation was carried out by drawing a straight line in the region of interest, as shown in Figure 4. From this region, the software calculated various parameters, including *R*_a_. This process was performed for images obtained at D0 and D45. Similarly, images obtained using the dermatoscope device were analyzed using the algorithms developed in this study to detect wrinkle regions and calculate *R*_a_, *R*_q_, and *W*_a_ for both D0 and D45 measurements.

### 2.6 Statistical analysis

The normality distribution of the obtained parameters was verified using the Shapiro–Wilk test.^31^ To determine whether significant statistical differences existed among different groups, the *T*‐test was applied to data with normal distribution, and the Wilcoxon test was applied to nonparametric data.^32^, ^33^

In addition, Spearman's correlation analysis was performed,^34^ and the correlation coefficient (*R*_s_) and probability of correlation (*p*‐value) were calculated. The *R*_s_ coefficient ranges from −1 to 1, indicating the strength of the correlation, which can be positive or negative. An *R*_s_ value from 0 to 0.19 indicates a very weak correlation, from 0.2 to 0.39 a weak correlation, from 0.4 to 0.69 a moderate correlation, from 0.7 to 0.89 a strong correlation, and from 0.9 to 1 a very strong correlation. The *p*‐value verifies the probability of accepting the null hypothesis, which assumes that there is no correlation between the datasets. A *p*‐value less than 0.05 rejects the null hypothesis and suggests a correlation between the datasets.

For all statistical analyses, a significance level of 95% was adopted.

## 3 RESULTS

**FIGURE 5:**
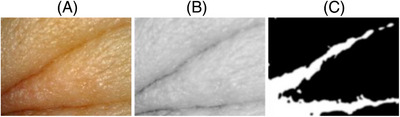
Image processing results: (A) Original image. (B) Gray‐scaled image with feature amplification applied. (C) Segmented image with wrinkles area in white.

The result of the wrinkle detection algorithm can be viewed in Figure 5, where the original image is presented on the left, the gray‐scaled features are amplified in the middle, and the segmented features are on the right.

**FIGURE 6:**
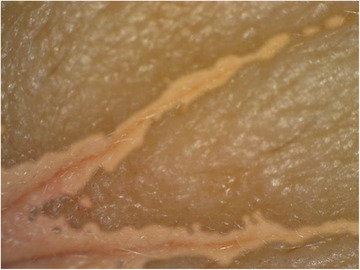
Segmented image superimposed on the original one, highlighting the areas with wrinkles.

To verify the points identified by the segmentation algorithm, the segmented image was superimposed onto the original image, as can be seen in Figure 6.

**FIGURE 7:**
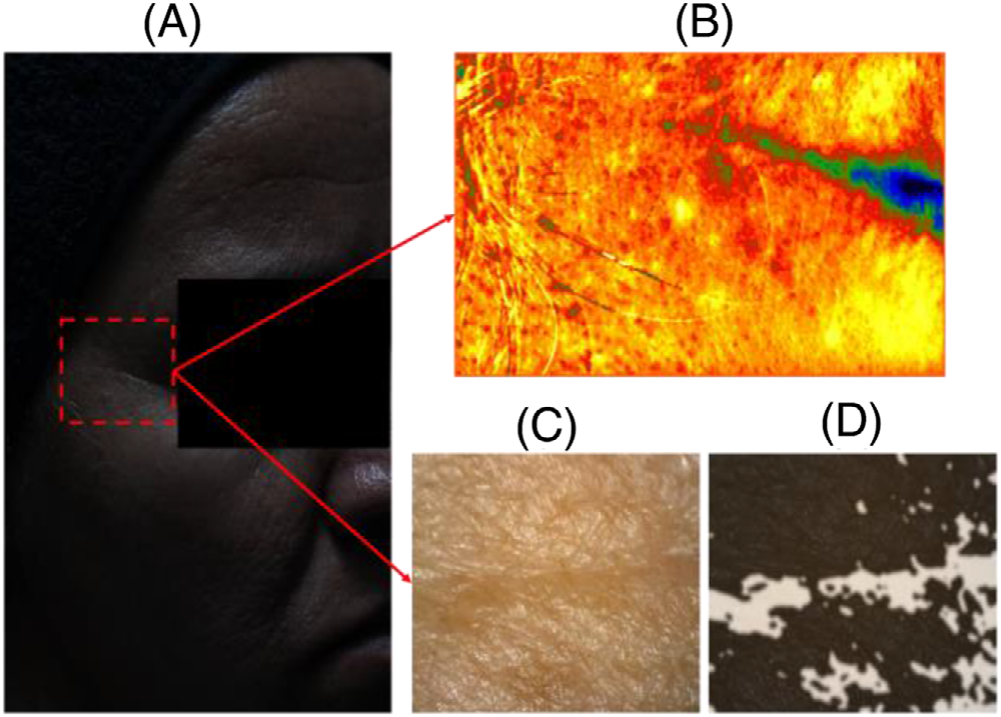
Illustration of the experiment: (A) Image of the right side of the research subject's face, with the periorbital area of interest highlighted in red. (B) Image obtained using PRIMOS. (C) Original image obtained using a dermatoscope. (D) Segmented image superimposed on the original one.

From the clinical study that was carried out, images of the periorbital region were obtained using two devices, the PRIMOS and the dermatoscope, as shown in Figure 7.

**FIGURE 8:**
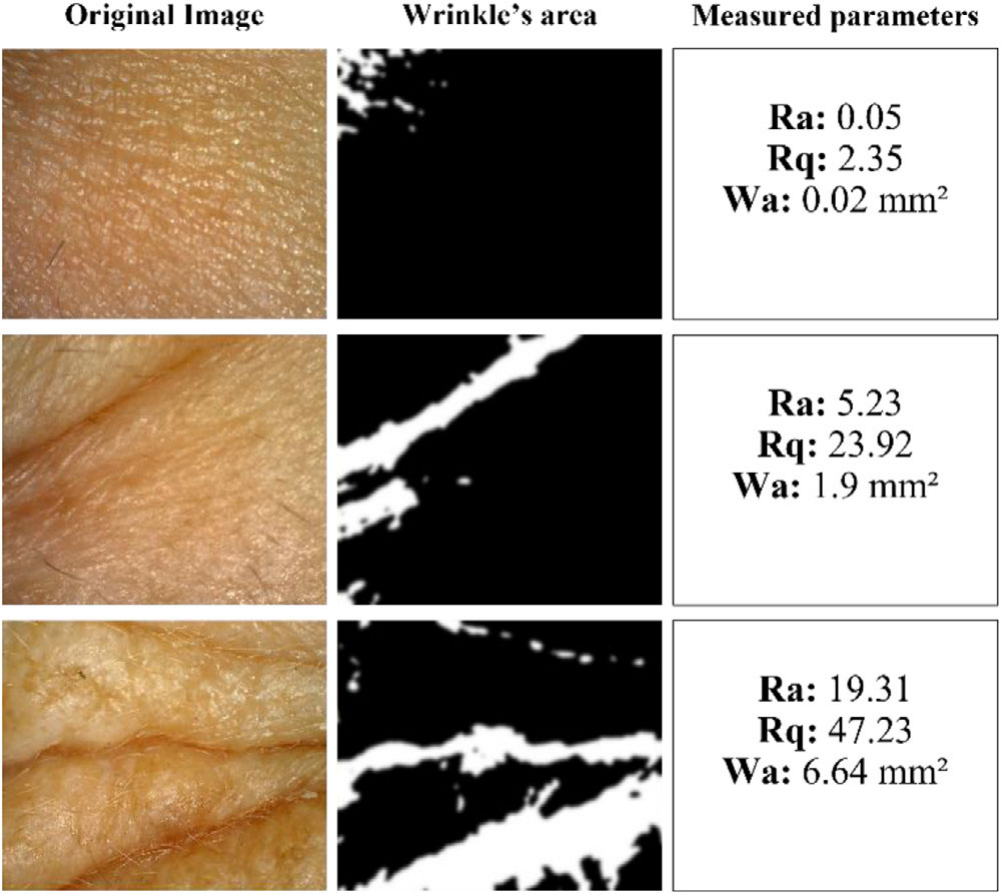
Some examples of dermatoscope images analyzed. From top to bottom, it is possible to visually identify the differences in wrinkle levels, which are evidenced by the calculated values.

In order to compare differences in wrinkles, Figure 8 displays images obtained from different levels of wrinkles, with the respective detection of wrinkle areas, as well as the calculated values.

From the obtained data, a normality test was performed for each dataset with the purpose of defining the statistical tests to be applied in the different comparisons. Table 2 shows the results of the Shapiro–Wilk test for each of the datasets. It is possible to verify that the *R*_a_ parameter obtained by both PRIMOS and the dermatoscope presented *p*‐values less than 0.05, indicating a non‐normal distribution, whereas the *R*_q_ parameter showed a normal distribution at both times.

**TABLE 2:** Shapiro–Wilk normality test.

**pa rameter**	***p*‐Value**
PRIMOS *R*_a_ D0	0.0071
PRIMOS *R*_a_ D45	0.0074
Dermatoscopy Ra D0	0.0024
Dermatoscopy *R*_a_ D45	0.0437
Dermatoscopy *R*_q_ D0	0.1049
Dermatoscopy *R*_q_ D45	0.3881

*Note*: *p*‐value less than 0.05 rejects the null hypothesis that the distribution is normal.

Spearman's correlation analysis was performed to determine the correlation between the values obtained by the device in comparison to PRIMOS at different times. When comparing the *R*_a_ values from D0, a weak correlation was found with a coefficient of *R*_s_ = 0.275 and a *p*‐value of 0.19, indicating no correlation. This low correlation may be due to the high standard deviation of the data in both measures. When evaluating the *R*_a_ parameter from the data obtained after 45 days, a moderate correlation was found with a coefficient of *R*_s_ = 0.47 and a *p*‐value of 0.018, providing strong evidence of correlation between the parameters. Similarly, the evaluation of the *R*_q_ parameter from the dermatoscope was carried out in comparison to the PRIMOS *R*_a_. At D0, a weak correlation was verified, with a coefficient of *R*_s_ = 0.276 and a *p*‐value of 0.19. For D45 data, a moderate correlation was obtained, with a coefficient of *R*_s_ = 0.466 and a *p*‐value of 0.02, indicating strong evidence of correlation between these two parameters.

**FIGURE 9:**
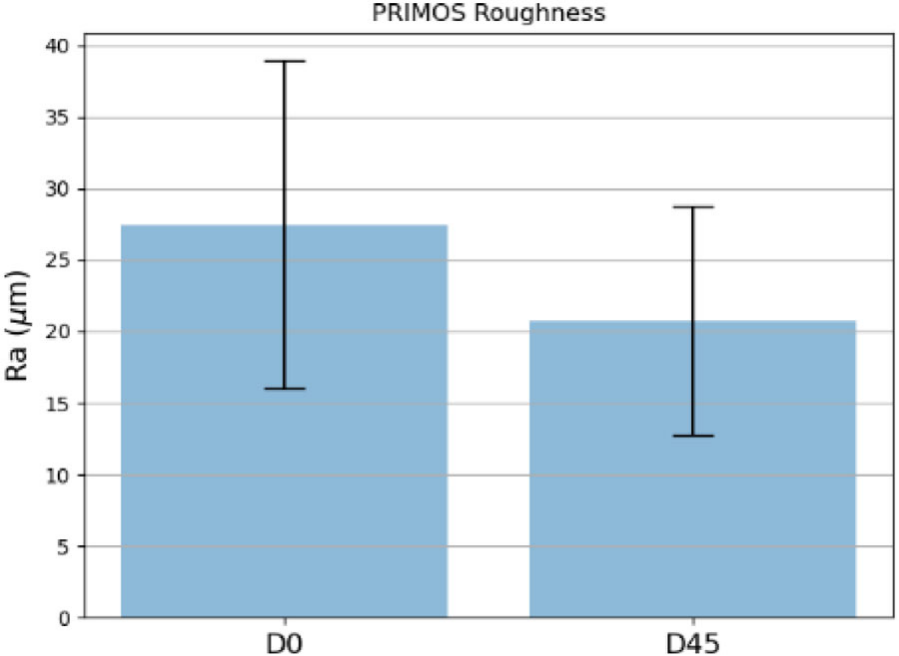
Parameter *R*_a_ obtained by the PRIMOS at times D0 and D45.

**FIGURE 10:**
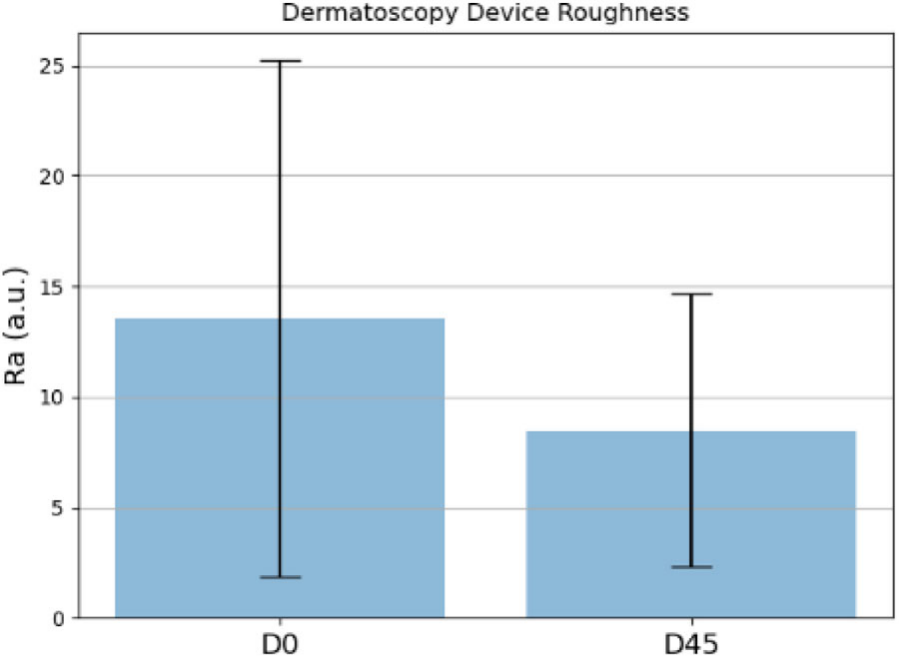
Parameter *R*_a_ obtained by the dermatoscope at times D0 and D45.

**FIGURE 11:**
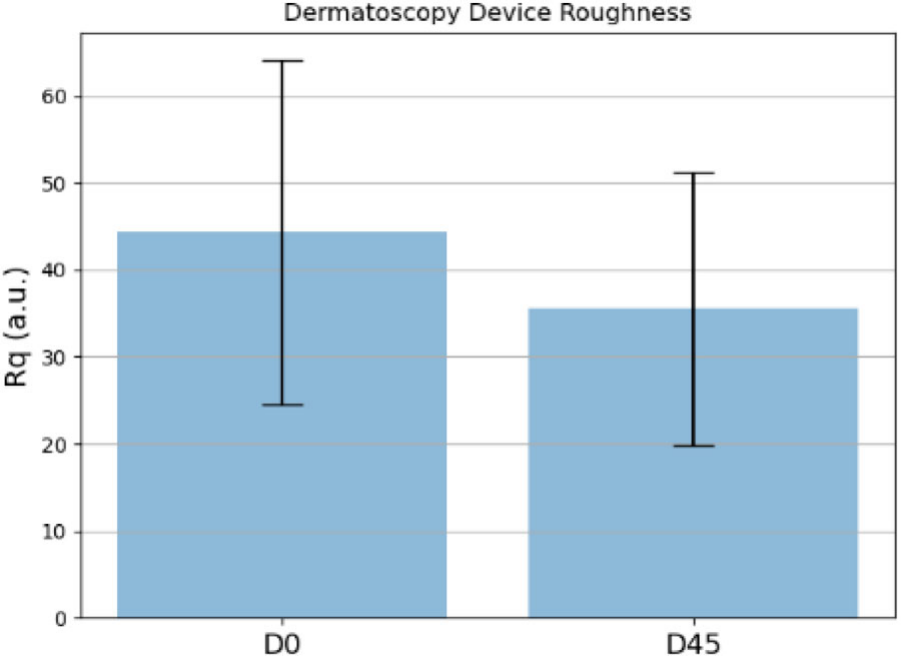
Parameter *R*_q_ obtained by the dermatoscope at times D0 and D45.

Figures 9, 10, 11 present a comparison between the two times for each of the parameters analyzed in bar charts. It is possible to observe a tendency of a decrease in the average roughness after 45 days of daily topical application of the dermocosmetic product.

The statistical analysis was performed to validate the effectiveness of the treatment by verifying the existence of statistical significance between the data obtained at time D0 and D45. The *R*_a_ parameter obtained by PRIMOS was analyzed using the Wilcoxon test, which resulted in a *p*‐value of 0.000000119. At a significance level of 95%, the null hypothesis of no difference after treatment can be rejected, indicating that there were statistically significant differences between D0 and D45 upon dermocosmetic topical application. The same analysis was performed for the *R*_a_ parameter from the dermatoscope images, resulting in a *p*‐value of 0.0229, which also detected statistically significant differences after using the dermocosmetic, assuming a significance level of 95%. By analyzing the *R*_a_ parameters from PRIMOS and dermatoscopy, it was found that the dermocosmetic was efficient in improving skin relief. Finally, the difference between the *R*_q_ values obtained at D0 and D45 was verified. As this dataset was the only one that presented normal distribution, *T*‐test was used for comparison. It resulted in a *p*‐value of 0.102, which, considering a 95% significance level, was unable to detect statistically significant differences between treatments.

## 4 CONCLUSION

In summary, data were collected using a dermatoscope device that provided maximized images focused on wrinkles, which were then compared to the gold standard device, PRIMOS. Along with the developed wrinkle detection algorithm and the new approach for roughness calculation, this becomes an interesting alternative for the analysis of skin relief, including the ability to detect significant statistical differences in the analysis of a dermatological treatment using a dermocosmetic. It is important to emphasize that in the analysis carried out with the PRIMOS system, a region of interest was demarcated, whereas in the proposed system, the analysis is carried out automatically. One of the great advantages of the proposed system is the low cost of the device, compared to established systems such as PRIMOS, VISIA, and VISIOSCAN. Thus, the proposed system becomes a viable option that provides quantitative values of skin quality to be used in dermatological clinics or clinical research institutions for evaluating the effectiveness of cosmetics or treatments.

## FUNDING INFORMATION

Coordination for the Improvement of Higher Level Personnel (CAPES), Grant Number: 88882.365434/2019‐01

## Data Availability

The data that support the findings of this study are available on request from the corresponding author.
